# Exploring emotional experience and word-of-mouth mechanisms in male-oriented live-action interactive romance games: a mixed-methods empirical study

**DOI:** 10.3389/fpsyg.2026.1849923

**Published:** 2026-06-26

**Authors:** Tang QinLi, Li Li

**Affiliations:** 1 School of Journalism and Communication, Anhui Broadcasting Movie and Television College, Hefei, Anhui, China; 2 Huace Film School, Zhejiang University of Media and Communications, Hangzhou, Zhejiang, China

**Keywords:** emotional experience, FMV games, live-action interactive romance games, mixed-methods research, parasocial relationships, sentiment analysis, user-generated content, word-of-mouth mechanism

## Abstract

Live-action interactive romance games gained widespread popularity in 2023, but there remains a lack of mechanistic research on how male-oriented emotional experiences are generated and transformed into word-of-mouth and consumption intentions. Centering on the questions of “why preferences arise and how positive reviews are formed,” this study selects player reviews of four representative works across multiple platforms as samples. Combining content analysis, emotional quantification, LDA topic modeling, and netnography, it sorts out five key elements—emotional interaction, narrative, gendered bodily representation, technology, and others—and their operational paths, thereby constructing the corresponding relationship between evaluation and impact. The results show that, at the work level, the intensity of emotional interaction is largely consistent with the ranking of ratings. Notably, this consistency holds only at this level, while the correlation between comment categories and individual scores is weak at the comment level. Narrative has the second strongest impact; gendered bodily representation and technical topics do not form a stable positive driving force, but instead weaken word-of-mouth in some contexts due to objectification or performance disputes. The conclusions provide actionable references for optimizing the emotional mechanism of film-game integrated works, operating male audiences, and managing word-of-mouth, and offer insights for content design, community maintenance, and commercial transformation.

## 1 Introduction

In the past few years, live-action interactive video games and interactive films have witnessed a rebirth both worldwide and within the Chinese Internet. They depend on “dynamic images + interactive interfaces” to structure branching narratives and real-time feedback. They constitute a steerable immersive viewing and gaming experience (Hanapiah and Nasir, 2024).

Research on interactive narrative and audience participation shows that audience participation is inscribed in the narrative behavior itself: interface navigation, feedback latency and the salience of branching consequences combine to shape emotional intensity and meaning-making (Caroux, 2023).

This paper explores the generation of word-of-mouth and secondary communication in romance-oriented interactive films and live-action interactive video games from the perspective of male audiences, as well as the interaction of emotional interaction, narrative organization, visual presentation, technical implementation and platform distribution. The short video ecosystem and platform-based distribution have enhanced the exposure and conversion efficiency of interactive texts, and the blended experience of film-like viewing and game-like control is reshaping the audience evaluation and recommendation logic (Hanapiah and Nasir, 2024). Meanwhile, the evidence on presence and related psychological mechanisms shows that the design aspects influence enjoyment and behavioral outcomes through the modulation of immersion and emotional engagement (Caroux, 2023).

The current research achievements mainly include the following three aspects:

(1) Interactive narrative and audience participation: Discussing the interaction rules of FMV (Full-Motion Video) interactive narratives, as well as audience interpretation, which provide direct insights into how participatory narratives and interface cues influence experiences (van de Ven, 2024).
(2) Identification and emotional involvement. Recent studies and quantitative evidence suggest that enjoyment, replay intention, word-of-mouth are related to players’ presence experience and investment (Caroux, 2023). (3)Visual cues and consumption: The theory of visual capitalism and cross-platform empirical study show that appearance and sexual cues can boost attention, trust and purchase intention, especially in highly visual platform scenarios (Djafarova and Bowes, 2021).

In the specific area of game media, the evidence for “whether sexualized presentation drives sales” is mixed and there have been designs and critical discourses against the male gaze (Valentowitsch, 2024). The recent literature shows that netnography is the analysis of the emotional cues of bullet comments and reviews, the norms of the platform and secondary creation collaboration through participatory observation in the original context, thus capturing the micro-mechanism of interactive images from the dimension of attention to emotional triggers and then to word-of-mouth diffusion. This has been validated in studies of localized collaboration and identity building in gaming and fan circle contexts, and has established operational processes and ethical principles for subcultural research (Kozinets and Gretzel, 2024).

As for the existing research findings, there are many problems to be solved urgently:

(1) FMV and interactive films are generally studied separately from dating simulations, and there is no joint word-of-mouth model that can simultaneously capture narrative branching, interface feedback and emotional engagement (Hanapiah and Nasir, 2024). (2) The absence of studies on the psychological and behavioral mechanisms of male-oriented samples, especially the absence of studies on the relationship between visual cues, emotional comfort and consumption behavior in the same model for joint testing (Lynch et al., 2024). (3) New information has been obtained on the current mechanisms of visual capital and beauty labor but has not been fully integrated into the audience mechanism model of interactive films (Xie et al., 2023).

It should be clarified from a unified framework of interactive story, audience emotion, and platform distribution what is the particular position and role of visual capital in the chain of word-of-mouth generation. To avoid conceptual fragmentation, visual cues should be incorporated into a unified analytical model together with variables such as story branching, emotional involvement and interface feedback into a single analytical model for assessing emotional effects. To improve the operability of the model, this study begins with the content and behavioral ends, divides the audience’s attention into five independent and parallel dimensions (emotional interaction, narrative, gendered bodily representation, technical indicators, and other comments), and then conducts detailed discussions combined with netnography.

The research object of this study is the male-oriented live-action interactive films/games. Data were collected through a netnographic research design from two main sources: first, online user comments on representative works were collected through web data crawling; second, original statements and contextual materials were collected through participatory observation in relevant online communities and supplementary online interviews. Twenty-five interviewees had played male-oriented live-action interactive romance games and had participated in related online discussion groups. Participants were selected based on their experience with gameplay, involvement in online groups and their ability to express their emotional experiences and word of mouth behaviors. The online interviews complemented the crawled comment data by offering deeper explanation of users’ emotional responses, engagement patterns and WOM reasons. The qualitative basis for netnographic analysis of this study consists of the crawling internet comments, community observations, and interview materials.

The research attempts to link content characteristics, audience emotions, and behavioral indicators in the same analytical framework to set up a testable word-of-mouth generation model for interactive films; and use contextual evidence provided by netnography to triangulate quantitative results, outputting actionable suggestions for emotional design, narrative organization, and platform operation.

## 2 Literature review

Interactive films and live-action interactive videos are text forms that take live-action images as bearers and allow explicit choices during playback. The choices of the audience causally influence the following shots or endings, so the viewer becomes a participant. The experience is no more a single path but a multi-path structure that may be explored (Koenitz, 2023). In this regard, content analysis is applied to obtain structured information with verifiable coding rules; emotional quantification translates text emotions to an ordinal scale for distribution, polarization, and difference testing, and can jointly show the co-occurrence relationship of themes and emotions with topic modeling (Liang and Chen, 2024); netnography focuses on participatory observation and reflexive recording in platform and community scenarios to explore the influence of expression norms and interaction rules on content production and retransmission (Padricelli and Punziano, 2023).

Research on interactive images in recent years can be summarized as three orientations. The first is qualitative and speculative, focusing on the branching structure and narrative interpretability, and discusses the relationship between key nodes, outcome diversity and audience understanding (Koenitz, 2023). The second is mainly quantitative research, targeting female-oriented interactive images, measuring psychological mechanisms such as role identification, parasocial relationships and intimacy construction to explain the differences in satisfaction and re-watching intention (Pei et al., 2025). The third is that the discussion on male-oriented interactive images is relatively outdated, and the research objects are mostly interactive texts from the early 21st century, almost all in the form of visual novels; the relevant analysis is mostly based on the visual cultural framework such as gendered gaze and gendered bodily representation, and the contextual testing is carried out through digital ethnography in interactive scenarios. And their explanatory power for contemporary platforms and multimodal engagement is limited due to the limitations of time and media format (Galbraith, 2011).

Based on previous research, existing studies have gradually reached a consensus on the impact of interaction structures on experiences and evaluations. Choice salience, time pressure, and branching differentiation are stably associated with immersion, empathy, and recommendation intention, and open endings and closed endings differ in replay attitudes (Mast et al., 2025). Clear prompts, timely feedback, visible outputs, branch visualization and process-based editing tools aid in maintaining narrative rhythm and structural consistency during content creation (Xu et al., 2025). At the platform level, recommendation mechanisms, pinning rules, and bullet comment norms serve to regulate emotional expression. At the data-method level, comment cleaning and vectorization, combined with ordinal emotion scales and LDA topic models, have become common methods, and the matrix presentation of themes and emotions is used to identify controversial focuses and group differences (Liang and Chen, 2024). For romance-oriented interactive images, qualitative methods are used to focus on role identification, parasocial relationships, and intimacy construction, pointing out that role attachment and relationship scripts can explain differences in satisfaction and rewatching intention, indicating the important role of key nodes and narrative interpretability in evaluation (Pei et al., 2025).

### 2.1 Research trends and findings

The overall research focus is moving from whether interaction is better to which interaction structure is more effective in various platform and community circumstances. Methodologically, the combined analysis of emotional quantification and topic modeling is becoming standard, and netnography is used to complement norms and contexts that are difficult to directly capture by mechanisms and indicators (Liang and Chen, 2024). In terms of tools, branch visualization and low-threshold creation systems have reduced the cost of tests and assessments (Xu et al., 2025). In romance-related texts, the reversibility of choices, interpretability of relationship scripts, and node salience are highly correlated with audience satisfaction, re-watching intention, and the role identification mechanism (Pei et al., 2025).

### 2.2 Shortcomings of existing literature

Many studies still classify differences according to whether there is interaction or multiple endings, and do not systematically compare countdowns, branch depth and outcome differentiation in the same framework, which limits the comparability of cross-work effects (Koenitz, 2023).

The lack of research on psychological and emotional differentiation at the platform context and community level. Existing research is predominantly focused on the rules and mechanisms at the platform level, such as pinning strategies, bullet comment norms, and review processes. However, the micro-processes of identity stratification, stance polarization, and emotional contagion in small-circle communities are not observed. Therefore, it is challenging to account for the variations in expressive intensity and stylistic characteristics among different communities on the same platform (Muñoz et al., 2024).

Lack of integration of quantitative indicators and interpretive frameworks: Typically, emotional evaluations and thematic identification are reported separately. Few studies combine interaction dynamics, emotional reactions and platform-specific contexts within the same dataset. This study constructs a theme-emotion matrix using a five-point emotional scale and LDA, and maps it to five-dimensional mechanism coding to identify mediating and moderating paths (Liang and Chen, 2024).

Absence of systematic evidence on male-oriented romantic emotional interactive images: The existing research mainly focuses on female-oriented and otome-oriented interactive texts, with limited quantitative characterization of substitution mechanisms, re-watching motivations, and outcome exploration of male audiences (Pei et al., 2025); The existing research on male audiences mainly employs visual novels as the research carrier and conducts case studies, lacking rigorous empirical research (Bouvard, 2025).

### 2.3 Research questions

*RQ1*: Is the audience’s overall preference for interactive films related to the five attention dimensions identified in the comments? Which dimension is more salient in relation to overall preference?

*RQ2*: Is the audience’s preference for interactive films mainly associated with emotional interaction? Compared with narrative, gendered bodily representation, technical indicators, and other comments, is emotional interaction more salient in audience evaluation?

## 3 Methods

This study adopts a mixed-methods approach combining five-dimensional content analysis, five-point emotional quantification, LDA topic modeling, and netnography. The empirical materials include crawled online user comments on representative male-oriented live-action interactive films/games, participatory observation records from relevant online communities, and supplementary online interviews with 25 informants.

### 3.1 Analytical framework and coding scheme

This study adopts a “mixed research” design, combining qualitative and quantitative analysis to examine audience responses to interactive images and interactive films. The qualitative part forms the coding ontology and operational definitions for comment analysis through contextual observation and contextual interpretation; on this basis, the quantitative part, as the core, focuses on five attention dimensions summarized from recent literature: ① Emotional interaction: Emphasizing the role of role identification, parasocial relationships, and emotional involvement in preferences and continuous participation (Schramm et al., 2024); ② Narrative: Focusing on the structural complexity of interactive narratives and audience agency, which has a key impact on immersive experience and emotional intensity (Hang and Azahari, 2023); ③Gendered bodily representation: Examining the potential impact of sexualized and objectified representations on audience evaluations and attitudes (Ferguson et al., 2022); ④ Technical indicators: Involving usability elements such as interaction logic, latency, branch salience, interface feedback, and audio-visual synchronization, which significantly shape experiences and evaluations (Brunnström et al., 2020); ⑤ Others: Incorporating factors such as platform mechanisms and content accessibility that have been identified in the literature as potentially affecting audience attitudes but are inconvenient to categorize separately, to avoid missing exogenous or cross-level variables (Sun et al., 2024).

The specific classification is shown in Table 1.

**Table 1:** Emotion classification table.

Emotion classification	Mainly focusing on the emotional interaction part in the works	Mainly focusing on the narrative part in the works	Mainly focusing on the display of gendered bodily representation	Mainly concerned about the technical indicators of the works	Other comments not belonging to the above four categories
Classification number	1	2	3	4	5

### 3.2 Methodological design, sampling procedure, and data

To address the research questions, this study adopts a mixed-methods design combining quantitative comment analysis with netnographic interpretation. Quantitative analysis was used to identify distributional, correlational, and semantic patterns in audience comments, while netnography was used to contextualize these patterns within online community discourse and platform practices.

The analytical framework of this study brings together three interrelated layers: the content features of the works, users’ emotional responses, and observable behavioral expressions in their comments. Rather than treating these layers separately, the study uses them to examine how audience evaluations are formed and articulated. Content features were specified through five dimensions derived from the literature: emotional interaction, narrative, gendered bodily representation, technical indicators, and other comments. Emotional responses were assessed with a five-point satisfaction scale, in which 5 represents “very satisfied” and 1 represents “very dissatisfied.” When comments contained identifiable references to continued playing, recommendation, payment, or word-of-mouth diffusion, these expressions were treated as behavioral indicators.

The empirical objects were romance-themed live-action interactive films or games that had generated relatively visible discussion on Chinese digital platforms. The selected works shared three basic features: they relied on live-action images and interactive choice mechanisms, they were organized around male-oriented romantic or emotional interaction, and they had accumulated user discussions on platforms such as Bilibili and Steam. The four selected works were the mainstream titles in this emerging market during the observation period, while other comparable works received limited market attention and did not generate enough public commentary for systematic analysis. Through web crawling, 1,471 comments were initially collected. After removing blank entries, meaningless symbols, advertisements, repeated items, and comments irrelevant to the selected works, 1,268 valid comments remained for subsequent analysis.

The coding of the valid comments proceeded along two lines. Each comment was first assigned an emotional score according to the five-point satisfaction scale, and then categorized under one primary label corresponding to the five attention dimensions. On this basis, the coded dataset was used for descriptive statistics, Pearson correlation analysis, and LDA topic modeling. The LDA model was applied to the cleaned corpus in order to identify recurring semantic patterns and high-weight word groups associated with emotional interaction, narrative experience, and other forms of audience evaluation.

To improve the reliability of the coding process, three independent experts were invited to participate in the classification work: one scholar in game studies, one researcher in digital media and communication, and one researcher specializing in computational text and sentiment analysis. All three coders worked with the same coding ontology and operational definitions. Inter-rater reliability was calculated before reconciliation, and the Krippendorff’s alpha reached 0.84, suggesting a high level of agreement. Cases of disagreement were then discussed collectively until a final coding decision was reached.

Netnographic materials were introduced to enrich and contextualize the quantitative findings. These materials consisted of public platform discussions, community posts, long-form reviews, social media interactions, and supplementary online interviews with 25 participants who had played male-oriented live-action interactive romance games and had taken part in related online discussions. The informants were purposively selected based on their gameplay experience and participation in relevant online discussions. Specifically, they were male users from Chinese-speaking regions where Simplified or Traditional Chinese is widely used, including Mainland China, Hong Kong, Macao, Taiwan, Malaysia, and Singapore. They had fully experienced at least one representative male-oriented live-action interactive romance work, with more than 30 h of gameplay, had participated in related online discussions, and were able to provide reflective descriptions of their emotional experiences, interaction practices, and word-of-mouth behaviors. Therefore, the term “users” in this study does not refer to an unrestricted global online community, but to a specific Chinese-language user group located within defined linguistic, regional, and platform-based boundaries. Accordingly, “community norms” are understood as the interaction conventions, evaluative vocabularies, affective expressions, and word-of-mouth practices formed within these Chinese-language online communities. The observation covered the period from June 2023 to August 2024. After screening, approximately 50,000 words of relevant textual material were retained. These materials were thematically coded around the five dimensions and compared with the quantitative results, including score distributions, correlation patterns, and LDA topics. By combining large-scale comment analysis with contextual interpretation, the study strengthens the transparency and replicability of its mixed-methods design.

## 4 Results: satisfaction distribution and topic modeling

### 4.1 Satisfaction score distribution and correlation analysis

**Figure 1:**
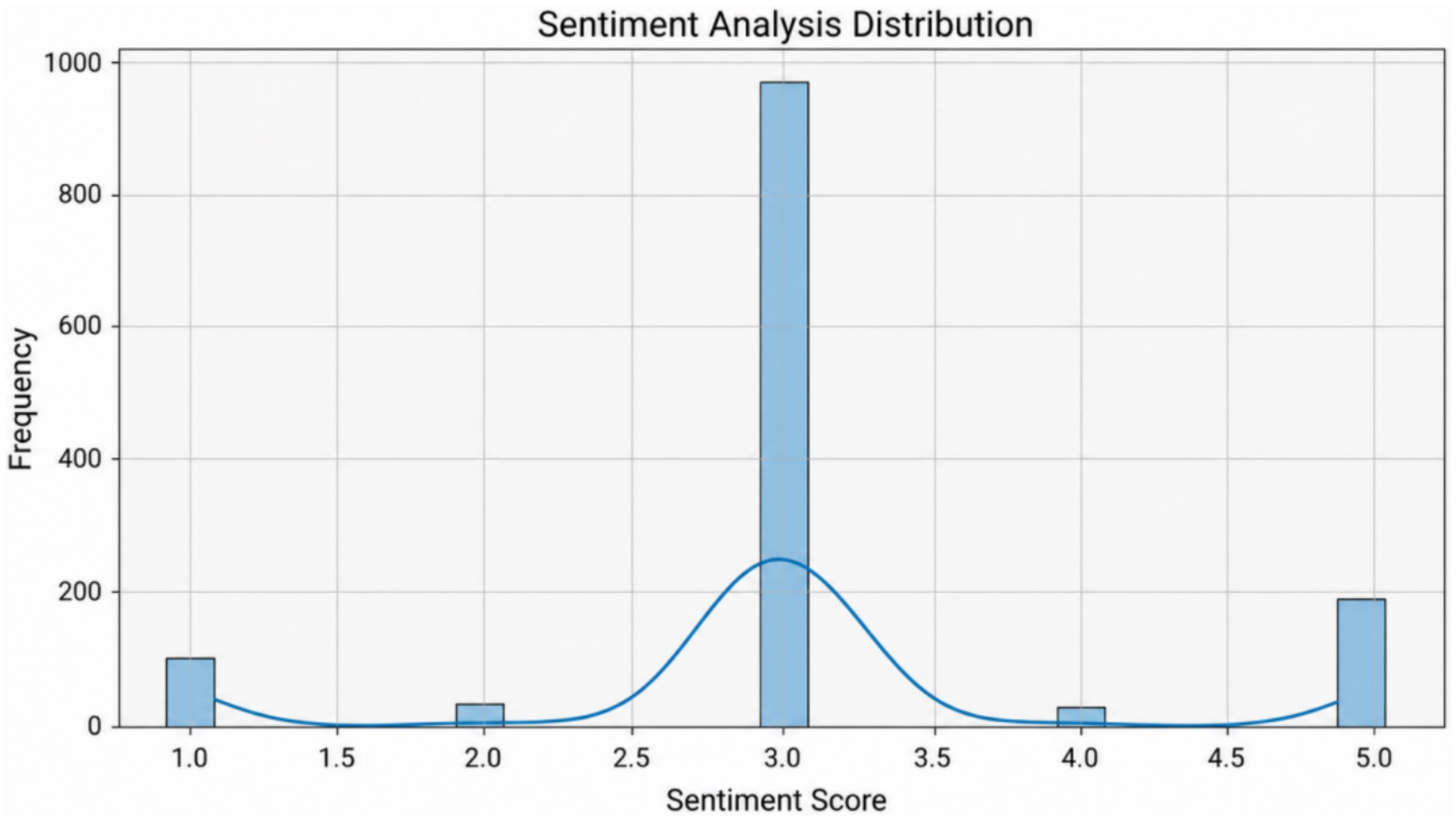
Sentiment analysis distribution.

For a more detailed study to reveal the relationship between specific work classifications and emotional scores, this study first conducts a normal distribution test, and the results are shown in Figure 1.

Many comments were scored at the neutral midpoint (Score 3). This concentration may have reduced the variation in satisfaction scores and contributed to the weak correlations found in the following analysis.

Based on Figure 1, descriptive statistics of the distribution of each work are obtained, as shown in Table 2.

**Table 2:** Descriptive statistics table.

Work title	Score 5	Score 4	Score 3	Score 2	Score 1	Valid N	Mean	Median	Mode
Tomorrow’s Love Puzzle	19	2	168	2	4	195	3.154	3	3
Knowledge, or Know Lady	101	19	376	9	5	510	3.396	3	3
Fell in Love with Coser	5	1	39	1	46	92	2.109	1.5	1
Love Is All Around	62	14	380	6	9	471	3.242	3	3
Total	187	36	963	18	64	1,268	3.208	3	3

The original Chinese titles corresponding to the four works are as follows: Tomorrow’s Love Puzzle (《拜托!明天和我恋爱吧》), Knowledge, or Know Lady (《美女, 请别影响我学习》), Fell in Love with Coser (《请做coser的主人》), and Love Is All Around (《完蛋!我被美女包围了!》). Higher scores indicate higher satisfaction: 5 = very satisfied and 1 = very dissatisfied. Valid N includes only records with complete emotion-classification and satisfaction-score fields.

Based on the score distribution in Table 2, it can be seen that “Knowledge, or Know Lady” has the highest mean value, indicating the most positive overall evaluation; “Love Is All Around” and “Tomorrow’s Love Puzzle” have mean values slightly above the neutral midpoint; while “Fell in Love with Coser” has the lowest mean value and a relatively high proportion of low scores, indicating the least positive overall evaluation. From a theoretical perspective: According to the emotional appraisal theory, differences in goal congruence, controllability, and novelty triggered by titles affect immediate emotions and overall scores. Role-playing and clear relationship scripts are more likely to cross the investment threshold and receive more positive evaluations, while generalized ambiguous settings mostly stay in the neutral range (Hamby and Jones, 2022); from the perspective of visual capitalism and attention economy, gendered bodily representation can bring short-term visibility, but under algorithm-driven repeated exposure, they are prone to trigger moral evaluations and asthetic fatigue (Vettehen and Schaap, 2023), which is consistent with the high proportion of Category 3 comments and the low mean score in “Fell in Love with Coser.” At the same time, the platform’s recommendation of physical elements and conflict cues will exacerbate polarization and negative feedback among young audiences, which is consistent with the polarization phenomenon shown in the data (Ringrose et al., 2024).

**Figure 2:**
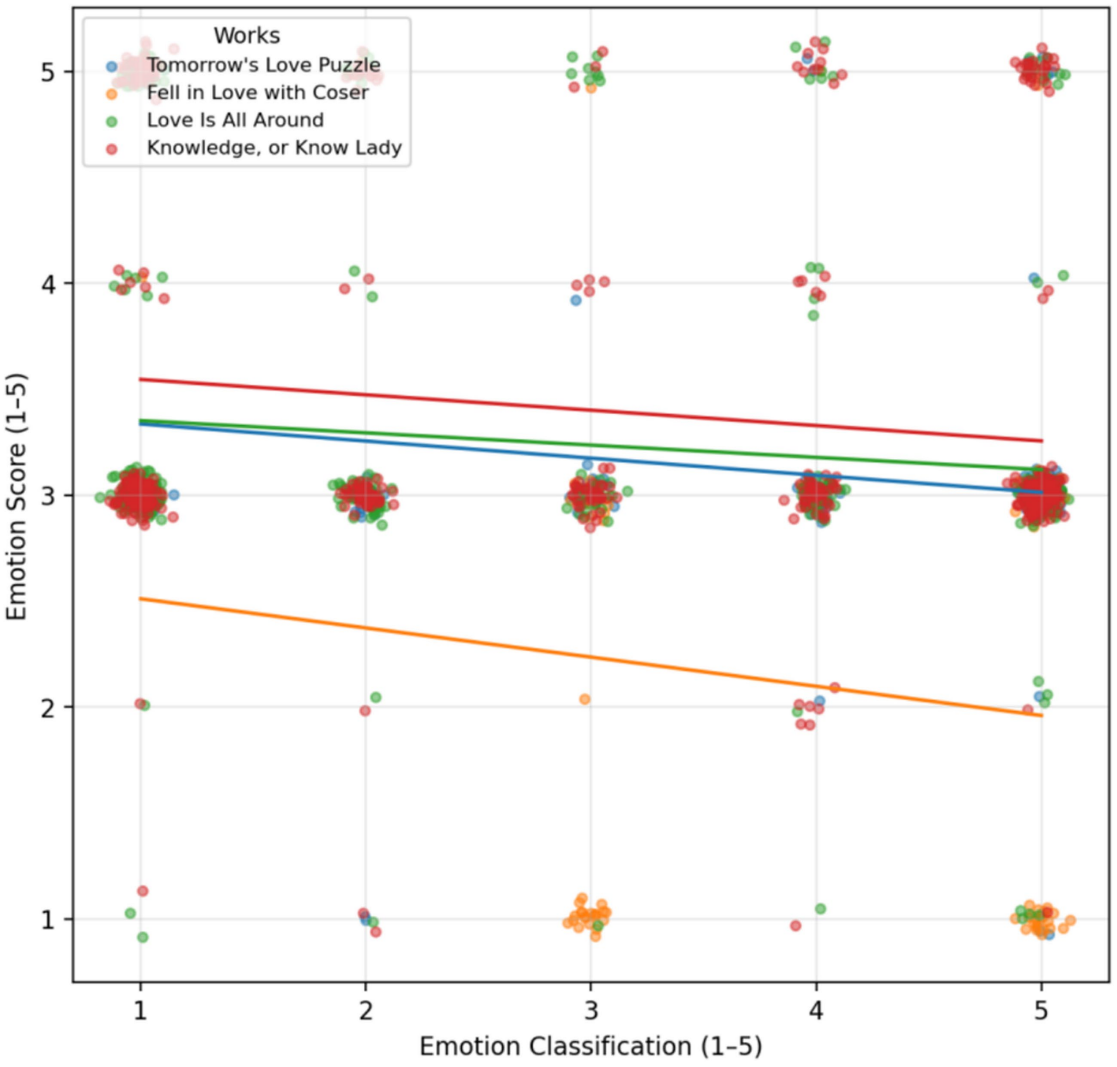
Pearson correlation between emotional classification and emotional scores across four works.

To test the correlation between emotional classification and emotional scores, Figure 2 (Pearson correlation coefficient graph) is drawn:

At the individual-comment level, Figure 2 indicates that the relationship between emotional classification and emotional scores varies across the four works. Pearson correlation is not significant at the 0.05 level in several cases. Where significance is observed the coefficients are small indicating that the association is weak rather than substantively strong. Taken together, these results suggest that the emotional classification alone is not enough to explain changes in emotional scores.

This weak association may be a reflection of the heterogenous nature of the audience responses. For some works, emotional classification may have little direct connection with scoring outcomes; for others, a limited relationship may still exist. This variation suggests that audience evaluation is affected by more than the emotional category assigned. From the perspective of emotional theory, users’ responses are also influenced by plot development, modes of emotional arousal, character construction, and individual affective states (Hamby and Jones, 2022). In this sense, emotional classification offers a useful analytical entry point, but cannot fully account for the factors driving audience scoring. In actual evaluation practice, viewers may be more sensitive to narrative arrangement, character appeal, or certain emotional moments, which explains the relatively weak correlation between classification and score to a certain extent.

**Figure 3:**
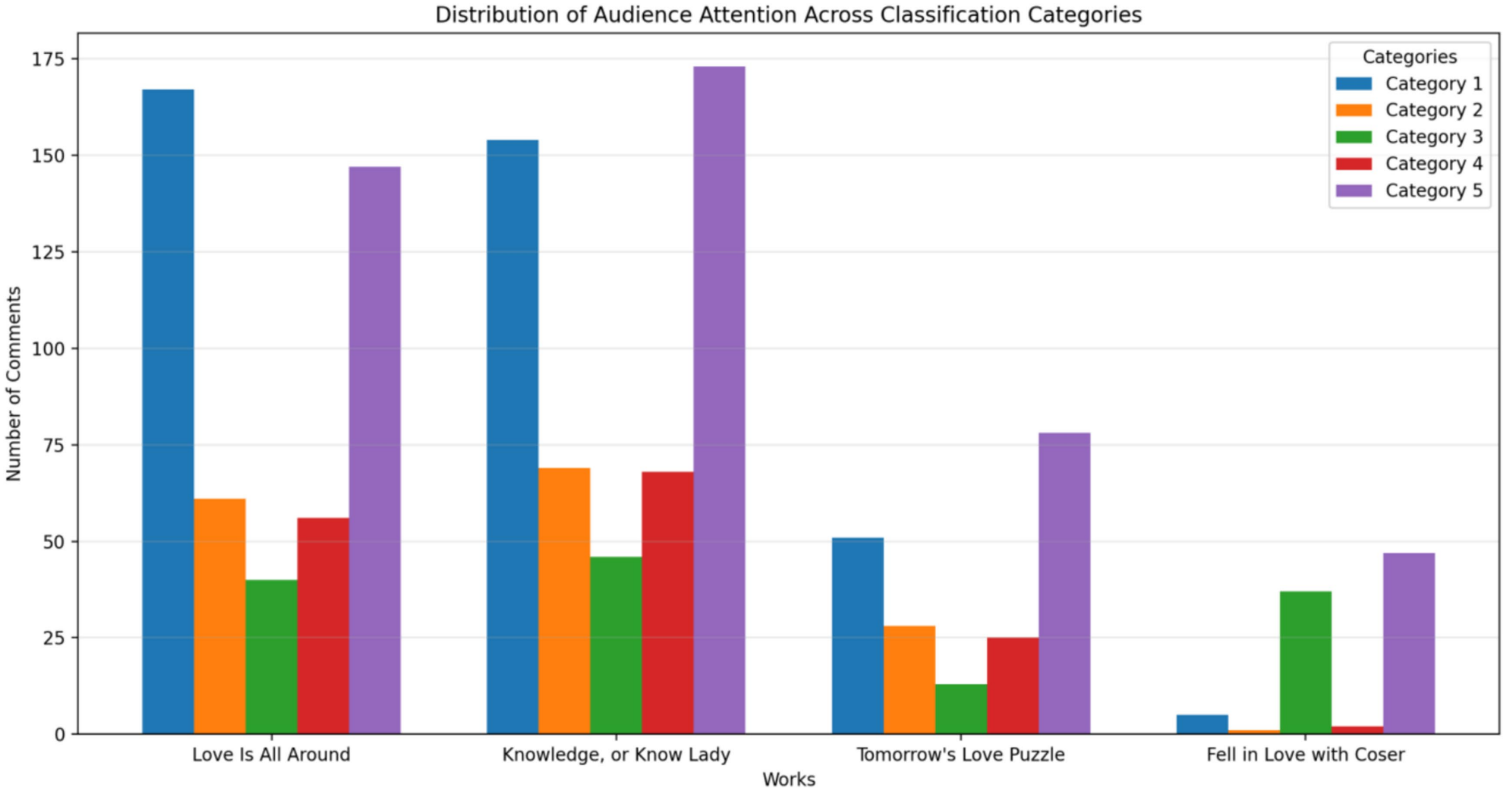
Distribution chart of works. The attached figure in the original text is a graph corresponding to work classification and quantity, which can be analyzed in combination with the data in the table.

Also, to better analyze the relation between work classification and emotional scores, Figure 3 is also introduced.

Through detailed data analysis, it is found that the emotional interaction part (Category 1) and the plot narrative part (Category 2) in the works have a greater impact on the audience’s emotional scores, while the display of gendered bodily representations (Category 3) and technical indicators (Category 4) have a loose relationship with the audience’s emotional scores. The specific analysis is as follows:

#### 4.1.1 Emotional interaction (Category 1): core driver of emotional needs

Category 1 (mainly focusing on the emotional interaction part in the works) is consistent with the order of emotional scores. This indicates that the audience highly evaluates emotional interaction elements; delicate emotional descriptions and real emotional communication can move the audience, meet their emotional needs, and make them experience emotional satisfaction and pleasure during viewing (Hamby and Jones, 2022); emotional interaction becomes the main reason why the audience likes the works because it can establish a deep connection with the audience at the emotional level.

#### 4.1.2 Narrative structure (Category 2): structural impact of story plots

The impact of Category 2 (mainly focusing on the narrative part in the works) on emotional scores is second only to emotional interaction. Although the number of people in Category 2 of “Knowledge, or know Lady” is slightly higher than that of “Love Is All Around,” in terms of the comment base, the proportion of Category 2 in both is almost the same. A good story plot and narrative structure can enhance the attractiveness of the work, strengthen the audience’s emotional investment, and thereby improve the audience satisfaction; the audience generally believes that a rigorous and engaging plot is more likely to trigger positive emotions, which is in line with most people’s viewing experience and evaluation standards (Ardjomandi, 2025).

#### 4.1.3 Display of gendered bodily representations (Category 3): short-term attention and long-term negative feedback

As a visual element, gendered bodily representations do not significantly improve audience satisfaction. Although they can attract the audience’s attention, they do not form strong emotional resonance or attachment (Vettehen and Schaap, 2023); some works fail to meet the audience’s emotional needs in other aspects, and comments focus on visual stimulation, forming a phenomenon of concentrated negative emotions (e.g., Fell in Love with Coser has the highest proportion of Category 3 comments and the lowest mean satisfaction score, indicating that concentrated attention to visual stimulation did not translate into positive overall evaluation; by contrast, Love Is All Around contains some Category 3 comments, but its higher mean score suggests that visual-related discussion did not dominate its overall evaluation).

#### 4.1.4 Technical indicators (Category 4): relative independence between technology and emotional experience

Category 4 (mainly focusing on the technical indicators of the works) has little impact on emotional scores. Although technical indicators (picture quality, sound effects, special effects, etc.) affect the viewing experience, they usually do not become the main driver of emotional scores; research shows that the improvement of technical quality increases audience satisfaction, but has a weak connection with emotional connection. Especially in highly interactive works, emotional responses are more dependent on emotional interaction and plot construction (Hamby and Jones, 2022).

Based on the above analysis, the audience’s emotional preference for interactive films is mainly driven by emotional interaction and narrative structure, while visual stimulation (especially the display of gendered bodily representations) has a negative impact on emotional scores and fails to effectively connect with the audience’s emotional needs. To further verify this conclusion, this study uses the LDA model to extract comment words, explores specific emotional responses under different emotional classifications, and comprehensively reveals the correlation between emotional interaction and audience emotional scores.

### 4.2 Topic modeling results

The LDA (Latent Dirichlet Allocation) model is a widely used topic model aimed at automatically mining potential topic structures from a large number of documents. Each topic consists of a set of words and their corresponding weights, where the weight reflects the importance of the word in a specific topic. Its core elements include: ① Topic: Identifying potential topics or content by clustering words in the document collection into multiple topics;

② Term: Each topic consists of several words that collectively describe the specific content of the topic; ③ Weight: Indicating the relative importance of the word in the topic; the higher the weight, the more core the role in defining the topic. In this way, LDA can assign one or more topics to each document, reveal the relationship between different topics, and provide a powerful tool for large-scale text data analysis (Blei et al., 2003).

Before confirming the LDA model, it is necessary to determine the number of topics (K). The meanings of three key indicators are as follows: ① Perplexity: Measuring the model’s predictive ability for held-out text; a lower value indicates better fitting, but it is not necessarily consistent with human interpretability (Blei et al., 2003); ② Topic_diversity@15: Calculating the proportion of unique words after combining the top 15 high-weight words of each topic; a higher proportion indicates clearer topic boundaries (Dieng et al., 2020); ③ Avg_topics_per_doc@0.1: Counting the number of topics with topic probability *θ* > 0.1 in each document and then taking the average, depicting the sparsity and mixing degree at the document level, which is affected by prior settings (Wallach et al., 2009).

#### 4.2.1 Determination of the number of topics (K)

The K value is calculated based on the original data (due to the limited number of original materials, the K value is tentatively set within 10):

*K* = 1, 2, 3: Too few topics; the two main axes of emotion and narrative are mixed, making it difficult to name the topic word list, which is inconsistent with the previous quantitative structure; *K* = 7, 8, 9, 10: Too many topics; under the conditions of this corpus and text length, emotion-related fragments are fragmented into similar topics, the overlap of high-frequency words between topics increases, interpretability decreases, and the mixing degree at the document level increases, making it difficult to align with the main axis of “emotion-led, narrative secondary”; *K* = 4, 5, 6: Quantitative comparison is shown in Table 3.

**Table 3:** K value comparison table.

K	Perplexity	Topic_diversity@15	Avg_topics_per_doc@0.1
4	592.5691181288134	0.7333333333333333	1.6184107002360346
5	579.8477964534229	0.68	1.6687647521636506
6	584.379579777899	0.7222222222222222	1.893784421715185

It can be seen from Table 3 that *K* = 5 is at the inflection point of topic diversity and perplexity: the perplexity of *K* = 4 (592.57) is higher than that of *K* = 5 (579.85), with poor fitting effect; although the topic diversity (0.733) is high, it compresses emotional and ending discussions into a few topics, resulting in insufficient separation of semantic boundaries and a narrow effective number of topics in documents (1.62); the perplexity of *K* = 6 (584.38) rebounds, with worse fitting than *K* = 5; the topic diversity (0.722) is close but semantic repetition occurs, and the effective number of topics in documents (1.89) is high with increased mixing degree. Considering the goodness of fit, topic differentiation, and document sparsity, *K* = 5 achieves the best balance with a combination of perplexity 579.85, topic diversity 0.68, and effective number of document topics 1.67. Therefore, *K* = 5 is finally determined.

#### 4.2.2 Analysis of LDA topic results

**Figure 4:**
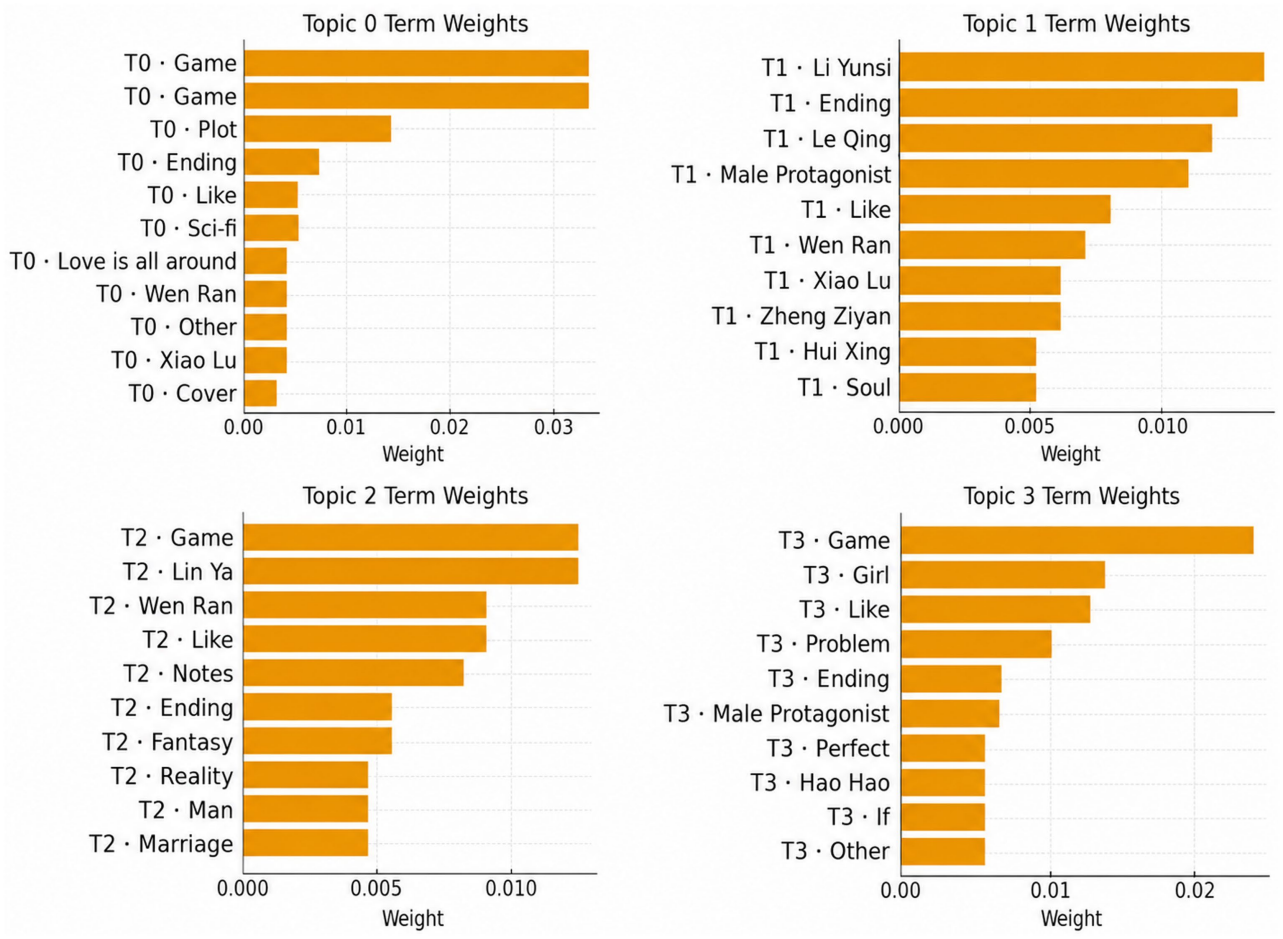
LDA classification and weight coefficient plot. The attached figure in the original text is a visual graph of the weight distribution of high-weight words under each topic, and the 5 topics can be analyzed in combination with word weights.

Based on the results generated by LDA, Figure 4 is drawn:

A preliminary analysis of the five divided Topics is as follows:

Within Topic 0, terms like “plot,” “ending,” “like,” “game,” and “sci-fi” are collected together. This indicates that the audience discussion in this topic is organized around the intersection between narrative structure and emotional interaction. In interactive texts, the emotional interaction must not be understood as a simple feedback loop between characters and players. It is embedded in the plot progression, the responses of the characters, and the design of the choice, all of which affect how players make decisions and how emotionally invested they are in the work (Carstensdottir et al., 2021). For this reason, narrative structure needs to maintain a certain degree of logical coherence, especially in relation to the consequences of player choices. As long as there is internal consistency to the story, the emotional investment is more likely to be stable. Accordingly, in this sense, the emotional interaction dimension is closely linked to players’ sense of agency and thus affects the players’ acceptance and evaluation of the ending (see Table 1).

Words in Topic 1 including “Li Yunsi” (a female protagonist/love interest in the work), “ending,” “comet,” and “soul” suggest discussion of character performance and emotional engagement. Characters in interactive texts are not only narrative devices, but also important vehicles for players’ affective responses. The emotional expression, behavioral logic, and quality of performance of the characters directly shape the players’ experiences, which in turn influence the way players interpret the characters and the overall story (de Lima et al., 2023). Previous research has demonstrated that the more emotionally invested, the more intense the evaluation of the ending tends to be. Thus, character performance is an important factor in the design of interactive texts, especially if the work aims to create sustained emotional engagement (see Table 1).

Topic 2 is characterized by keywords such as “like,” “Wen Ran” (a female protagonist/love interest in the work), and “Lin Ya” (a female protagonist/love interest in the work). These terms indicate a stronger emphasis on intimate emotional interaction between characters and players, as well as on the emotional guidance embedded in the narrative. In this context, emotional interaction depends not only on providing choices and feedback, but also whether the emotional trajectories of the characters match the players’ expectations. In many of the interactive fiction games character development is often closely linked to plot development. It is not just an external element of the storyline, but a mechanism of emotional identification of the players (Borkovich, 2022). As players become more invested in the plot, emotional interaction with the story world increases (see Table 1).

Topic 3 keyword cluster “girls,” “male protagonist,” “perfect” and “problem” show the relation between gender representation and emotional interaction. Gendered character design affects not only the fundamental design of game roles, but also the organization and interpretation of emotional interaction. The gender identity and emotional performance of characters affect players’ understanding of character behavior, evaluation of the consequences of decision-making, and maintenance of emotional involvement (Carstensdottir et al., 2021). In this respect, gendered settings do more than shape the emotional choices of players; they also provide a cognitive framework through which players understand character actions, relational dynamics, and narrative turns (see Table 1).

There are also “ending,” “acting skills” and “teacher” in topic 4, which indicates the other association of this topic is more related to technical evaluation, performance quality and narrative consistency. In live-action interactive works acting, performance details and emotional expression are an important basis for players’ affective responses. Simultaneously, a key criterion for assessing the credibility of an ending is the perceived consistency between character behavior and plot development. de Lima et al. (2023) point out that the coordination of technical design and character performance may affect players’ recognition of the ending, and the recognition may also feed back to emotional investment. Thus technical execution and quality of performance are not extraneous to emotional experience, but are part of the construction of narrative believability and affective identification (see Table 1).

These five topics broadly align with the previous classification and provide semantic support for the coding framework.

#### 4.2.3 Joint detection and summary of high-frequency words

On the basis of the five topics, further joint detection of high-frequency words is conducted (screening standard: occurrence frequency ≥ 2), and the results are shown in Table 4:

**Table 4:** High-frequency word distribution table.

Term_EN	Topic 0	Topic 1	Topic 2	Topic 3	Topic 4
Ending	0.007	0.013	0.006	0.006	0.012
Game	0.034		0.014	0.024	0.01
Like	0.005	0.008	0.01	0.012	
Love is all around	0.004				0.008
Male protagonist		0.011		0.006	
Other	0.004			0.005	
Wen Ran	0.004	0.007	0.01		
Xiao Lu	0.004	0.006			

Based on Table 4 and Figure 4, the summary analysis is as follows:

The current distribution of high-frequency words shows that the semantic focus mainly lies on the two ends of emotion and narrative: the emotional dimension focuses on the names of popular female characters (such as “Wen Ran” and “Xiao Lu”) and references such as “male protagonist,” revealing the audience’s attachment, substitution, and preference for characters; the narrative dimension is reflected in the continuous discussion of “ending,” indicating that evaluation and communication return to story closure and meaning judgment. Overall, the occurrence frequency of emotion-related words is significantly higher than that of narrative-related words, which is consistent with the previous quantitative conclusion (emotional interaction is more salient in relation to scores and likes/dislikes, followed by narrative factors).

In general, the analysis of topics shows that in interactive texts emotional interaction, narrative structure, character representation and technical realization do not act as separate dimensions. They are deeply intertwined in how they affect players’ sense of participation and their emotional responses. Of these elements, emotional interaction and narrative structure seem to be particularly important. Choices and feedback organize the player’s emotional process, while coherent plots and reasonable character reactions help maintain immersion, agency and role substitution. Some works have female physical elements but the whole weight of them in the LDA results is relatively small. This suggests that emotional depth and narrative coherence are more likely to shape players’ decisions and affective responses than gendered visual representation. The findings further emphasize the importance of emotion and narrative in the interactive design and audience experience.

This analytical framework also provides the foundation for the netnographic investigation that follows. The quantitative analysis has already shown the importance of emotional interaction and narrative structure, the netnographic part focuses on the social and discursive contexts in which these elements are discussed, expanded and reinterpreted by the players. In particular, it looks at how social platforms’ interactions intensify emotional resonance, make certain characters or plotlines more visible in the discourse, and encourage community engagement in the secondary creation and collective interpretation of interactive texts.

## 5 Netnography

The purpose of this paper is to explore the generation and evolution of interaction, emotion and community identification on social platforms based on netnography. For this part of the analysis, the materials are audience interview data, and long-term observations of online communities like Steam, YouTube and Twitter. The study collects comments, discussions, feedback and user-generated content from these platforms in order to track the ways in which interactive texts are circulated, discussed and re-created within digital communities.

Netnography has also been used to examine digitally mediated co-play and learning interactions in online game/video contexts, showing its suitability for studying interaction, participation, and meaning-making in platform-based media practices (Toh and Lim, 2023).

The observation period was between June 2023 and August 2024. During this period the research team regularly gathered data, observed platform interactions and interviewed audiences. These materials enabled us to capture changes in community dynamics and psychological responses of users over time. Initially, a lot of text and audio was collected. After screening, approximately 50,000 words of effective original text were retained. These materials included user comments, forum posts, long reviews and social media interactions. The selection criteria were relevance to research questions, especially materials on emotional interaction, community identification and group discussion. Then, the retained data were ordered in time so that the changes in audience behavior, emotional fluctuation, identity construction and group identification could be studied as part of an ongoing interaction process.

At the analysis stage, thematic analysis was used to investigate in depth the digital field notes and community interaction data (Braun and Clarke, 2006). The netnographic analysis builds on the findings of the preceding quantitative sections, which had already identified and classified the main topics, rather than starting from a completely separate framework. It emphasizes two main axes—emotion and narrative—but also attends to the ways in which technology and gendered bodily representation are taken up in audience talk and play a role in interpretive practices.

### 5.1 Emotional level

The audience first shows love for specific characters, such as: “Wang Yiman is truly ideal! She is really sunny, and the actor’s appearance and style are quite androgynous—both dominant and cute!” In the process of showing specific love, audiences with the same views will form character communities over time (about 1–2 months), and the topics will gradually develop toward romance, such as: “Wen Ran is really charming; it would be great to have a girlfriend like her in real life.” “I also want to fall in love with Wen Ran.” Many audiences focus on romantic details, such as: “I found that the most touching thing is not Chu Chu’s tears or smiles, but when she gave me pork rib soup, she did not let me use a disposable spoon.”

Subsequently, some audiences begin to express emotional suppression, such as: “Everything in the game is a fantasy; in real life, I’m just an otaku, and no one loves me.” “This kind of romance is probably a plot from a fantasy novel.” In the initial stage, a small number of audiences capture the gaze perspective, such as: “There are many close-up shots of Teacher Ouyang with clear intentions” (the audience clicked “dislike”), “Lin Yueqing’s figure is so good that no one can resist.” However, as the game has been released for a period of time (usually 2 months), most audiences show obvious negative emotions toward the gendered bodily representation, such as: “Emotional communication is more important than edge-ball content.” “We know it is industrial sugar, but you cannot force-feed it; all the plots inside are the most clichéd and vulgar ones.”

From a theoretical perspective: The audience’s love for specific characters is usually based on appearance, personality, and actor performance, which can be explained by the “emotional attraction” theory (Hipson and Mohammad, 2021); over time, audiences with the same views form character communities, and discussions gradually turn to romantic topics, with emotional investment amplified by group interaction. At the same time, some audiences express emotional suppression (such as comparing idealized relationships with real situations), and this tendency to buffer real shortages through virtual situations can be defined as the “emotional escape” mechanism (Marques et al., 2023).

In terms of gender presentation, early discussions are more likely to focus on “gaze” and physical close-ups, but when the release time is extended (about 2 months), negative evaluations are more concentrated on the rejection of “edge-ball content” and “clichéd vulgar plots,” showing that the audience’s requirements for emotional authenticity and narrative consistency are higher than their tolerance for gendered bodily representations; this shift is consistent with the compensatory media use framework, that is, when media content fails to meet higher-level emotional and narrative expectations, the audience will adjust discomfort through critical discourse or avoidance (Li and Zhuo, 2023).

### 5.2 Narrative level

At the narrative level, the audience’s comments mainly focus on three aspects:

Evaluation of plot logic: Such comments are the most common and show obvious polarization. For favorite works, the audience’s evaluations are full of praise, such as: “This one is better in terms of setting—it is slightly more reasonable, not so sci-fi, and focuses on logic; secondly, the script and lines are well-designed, not so awkward. Most of the character settings are classmates who already know each other, which is a good setting, avoiding the difficulty of female characters being overly familiar at the beginning.” For disliked works, the audience directly criticizes the logic of the plot, such as: “What is with this plot. It is disjointed and illogical; each unit has no connection, and it feels inexplicable. When I watched the second unit, there was a sudden competition and a sudden dinner together.” It can be seen that the plot logic affects the audience’s views on characters, thereby directly acting on the emotional level.

Completeness of narrative structure: The audience generally takes the completeness of the narrative structure for granted; therefore, when the work has a complete narrative structure, the audience will not praise it specially; on the contrary, for works with incomplete narrative structures, the audience expresses obvious discomfort, such as: “The process is a bit short, the chapters jump a bit fast, and the ending is too hasty.” “The endings of several storylines are forced; it feels like the story is not over but it actually is.”

Impact of technology on narrative: The audience mainly mentions the negative impact of technical design on the narrative experience (technical flaws reduce immersion), such as: “I’ve gone through all the plots, but it is stuck at 96%. The same with Chapter 6—I’ve gone through all the plots, all the endings including success, failure, and being single, and the one with 7 people, but it is still stuck at 99%. Can anyone summarize all the parts where the plot cannot be skipped? I guess I must be stuck on a few non-skippable plots that prevent me from getting 100%.” Another audience mentioned: “The dubbing is a bit distracting; the audio- visual asynchrony makes it impossible for me to immerse myself in the plot.” The interviewed audiences generally said that such interactive films are not blockbuster special effects or AAA games, so their expectations for special effects and grand scenes are low; technically, it only needs to be smooth and bug-free; therefore, the audience did not praise the technical aspect, and almost all the comments made are criticisms. If no technical issues are mentioned, it may mean that the game’s technical aspect is basically problem-free.

In addition, as the number of such works increases, the audience’s pickiness increases day by day; it can be found that the later the comments and interview content are on the timeline, the more biased they are toward negative evaluations.

The audience’s narrative evaluations can be summarized into a coherent evaluation mechanism: when the audience perceives that the causal connections are complete, foreshadowing is resolved, and the ending is properly concluded, narrative closure is established, and positive evaluations are more likely to appear; once problems such as “disjointedness,” “no connection between units,” and “hasty conclusion” occur, the closure is broken, and negative feedback follows, which gradually accumulates into stronger disappointment in subsequent comments and interviews (Krakowiak, 2024).

This set of reactions does not only come from the text level; the media implementation layer also intervenes in the evaluation: the narrative of interactive works relies on an available system and a stable presentation process. Technical breakpoints such as progress stuck, audio-visual asynchrony, and distracting dubbing will split the coherence of the timeline and event causality, thereby weakening understanding and emotional following; even if the audience has low technical expectations for non-AAA or non-special effects blockbusters, technical failures will amplify their interference with the narrative experience through “reverse marking” (Koenitz et al., 2022).

At the same time, the polarization in the comment section is not accidental, but the result of “active modeling” as described in cognitive narratology: different audiences integrate clues, infer motivations, and connect events according to their own experiences and tolerance thresholds. When clues are sparse or connections are abrupt, differences in reasoning strategies are more likely to differentiate into opposite judgments of “logical” and “illogical,” which are strengthened and solidified in community interactions (Piper et al., 2021). In addition, narrative quality directly affects the emotional level, especially the audience’s perception of characters.

### 5.3 Concept summary

**Figure 5:**
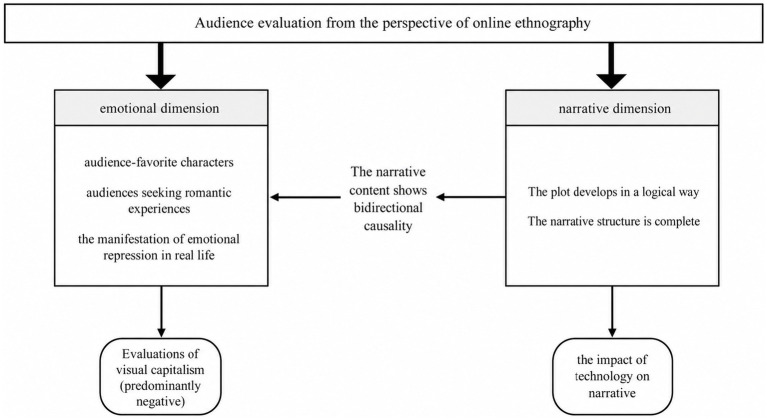
Concept map of digital ethnography. The attached figure in the original text shows that from the netnographic perspective, the audience’s evaluation is the result of the joint action of the plot logic structure at the narrative level, the impact of technology on the narrative, and the character love, immersive experience, and real emotional projection at the emotional level, and the narrative directly affects the emotion.

Based on the previous netnographic analysis, Conceptual Figure 5 is drawn:

Combined with Figure 5 and netnographic analysis, it can be seen that the previous quantitative research and netnography mutually verify, further proving that emotional interaction and narrative structure are the main axes of audience evaluation; at the same time, on the basis of quantitative research, it further explores the specific details of elements such as emotion and narrative (such as the emotional causes and specific feelings of the audience toward characters). The impact of gendered bodily representation is time-dependent, while technology mostly intervenes in the form of “defects triggering negative reviews”; the overall mechanism of narrative is: causal clues and closure enhance emotional recognition, while logical breaks and technical interruptions quickly transform into negative word-of-mouth. Specifically, it can be summarized into four points:

The root cause of emotion and narrative forming the main axis of evaluation: The audience calibrates emotional investment with “causal closure”—when clues, motivations, and results can be integrated into a self-consistent chain, perceived uncertainty may be reduced. Emotional rewards are fulfilled, and character preferences are consolidated; once integration fails, unrecovered foreshadowing and abrupt turns will amplify uncertainty, and emotional investment will turn negative.

The law of topic migration in community discussions: Community discussions migrate from “character preferences” to “romantic discussions and logical reviews” within 1–2 months, which stems from the gradual generation and rising threshold of group norms—initially uniting the group with emotional resonance, and then shifting the focus of evaluation to debatable narrative evidence to maintain group differentiation and discourse power, leading to increasing requirements for causal consistency and structural completeness.

The attention attenuation mechanism of gendered bodily representation: The attenuation of attention to gendered bodily representation over time may be interpreted as a shift in salience and value ordering—initial novelty stimulation increases discussion heat, and as the plot and character relationships become denser, the audience shifts evaluation resources to semantic and structural dimensions that can explain emotional rewards; moreover, in public discussions, moral and esthetic norms will label “edge-ball” narratives as low-information and low-reputation-gain strategies, thereby depreciating them.

The negative intervention path of technical factors: Technical factors mostly enter the discourse in the form of “defects,” which stems from the combined effect of “default expectations” and “attribution paths”—in non-AAA scenarios, technology is regarded as an “invisible infrastructure,” and smooth operation does not generate reportable signals; once lag or asynchrony occurs, the audience will attribute the understanding interruption to the media layer, making it difficult to maintain the continuity of the narrative causal chain, and negative emotions converge into a word-of-mouth inflection point through communicable “break events.”

## 6 Discussion

This study examined male-oriented live-action interactive films by linking content elements, emotional responses, and audience evaluations within a unified analytical framework. Through content analysis, emotional quantification, Pearson correlation analysis, LDA topic modeling, and netnographic interpretation, the study explored how audiences form preferences toward this emerging type of interactive audiovisual media.

### 6.1 Theoretical contributions

The main theoretical contribution of this study is the construction of a dual core model of emotion and narrative from a netnographic perspective. This model is not to be read as a causal model already statistically verified. Instead, it is a theory-building framework derived from online audience discourse and supported by multiple lines of empirical evidence, including content analysis, emotional quantification, correlation analysis, and LDA topic modeling.

The model’s strength is in connecting dimensions that are usually studied separately in interactive media research. Audience evaluation is not seen here as a composite of unrelated variables such as interaction structure, emotional involvement, visual presentation and narrative experience but rather as a function of their interrelationship. These empirical results are the foundation for this theoretical integration.

Table 2 shows the differences in satisfaction among the selected works. At the individual-comment level, Figure 2 shows that the correlation between emotional classification and emotional scores is generally low. Figure 3 shows the distribution of audience attention in classification categories. Table 4 and Figure 4 show that the main topic terms are concentrated in emotional preference, ending, plot development, and character relationships. Therefore, the dual-core theoretical model of emotion and narrative should be understood as an interpretive model constructed through netnographic analysis and supported by quantitative and topic-modeling evidence. It provides a framework for explaining how emotional interaction and narrative structure are jointly associated with audience preference and word-of-mouth evaluation in male-oriented live-action interactive films. Future research may further test this model through larger samples, regression-based analysis, structural equation modeling, or experimental designs.

### 6.2 Practical implications

The research shows that the key to the creation and operation of interactive films lies in three points: (1) Strengthen the credibility and emotional authenticity of character relationships; (2) Maintain the coherence of the narrative causal chain and stable rhythm; (3) Reasonably use sexual cues (although they can attract attention initially, emotional resonance and narrative depth are needed as support for continuous communication and retention).

In this context, technical design should focus on operational fluency and responsive feedback. If the setup is working well and interaction is flowing and the system is responding in a timely fashion, the media layer is less likely to disrupt the narrative flow. This helps to maintain immersion, and allows emotional investment to develop without being constantly interrupted by technical friction.

### 6.3 Limitations and future directions of research

Several limitations of this study must be acknowledged. The first relates to the nature of the data set. The analysis is mostly based on comments made by users of online platforms, and therefore the sample may over-represent users with somewhat strong attitudes. The commenters are the ones who choose to comment, and usually the ones with clear negative or positive opinions. The silent viewers or casual players or those with mild opinions are not likely to be present in the data. Therefore, the results should be interpreted with caution and not as representing the entire population of viewers of male-oriented live-action interactive films.

Another limitation is the linguistic and cultural scope of the data. Most of the comments studied in this article are from digital platforms in Chinese language, while part of the theoretical framework is based on English-language scholarship on interactive media, parasocial interaction, and digital narrative. Although the analysis is conducted with the aim of maintaining the original cultural and linguistic context of the comments, some platform-specific expressions, local terms and culturally embedded meanings may not be fully captured when interpreted through concepts developed in a different academic context. Future research may also investigate the generalizability of the proposed framework across languages and cultures.

The study is also limited by the type and time span of the materials. Online comments are useful to gage audience perception but provide only a limited picture of user experience. They are not able to fully capture the change in preferences over a longer period, the actual use of the works by the viewers, whether they continue to play, or the development of payment behavior. This study suggests some differences among male audiences, such as differences in their gaming experience and media-use habits. However, these differences still need more in-depth psychological and behavioral tracking. A further limitation is that many comments fall at the midpoint of the five-point scale. This may have narrowed the spread of scores and made the correlations appear weaker. The scale was useful for keeping the coding consistent across comments, but future studies could use finer-grained measures or combine ratings with behavioral indicators to capture audience differences more fully.

Finally, the ecological validity of the dual-core theoretical model of emotion and narrative should be further investigated. The model proposed here is developed through netnographic interpretation and is supported by descriptive statistics, correlation analysis and LDA topic modeling. However, it has not yet been validated in longitudinal observation, experimental research, regression-based analysis, or structural equation modeling. Thus, it is to be viewed as an interpretive theoretical framework, not as a fully verified explanatory model. This framework can be subject to more systematic empirical testing in future research.

Future research can proceed in three directions. First, the sample scope and time span should be expanded by including more platforms, longer observation periods, and cross-cultural datasets. Second, future studies can introduce multimodal data, such as video images, sound design, performance style, and interface interaction, to better capture the audiovisual characteristics of live-action interactive films. Third, the subgroup classification of male audiences should be refined by considering variables such as age, game experience, platform use, viewing motivation, and payment history, so as to test whether the effects of emotion and narrative differ across audience groups.

### 6.4 Connection with existing literature

The research results are consistent with the main viewpoints of existing literature: it further verifies the conclusion that “immersion and emotional involvement improve satisfaction and replay intention” (Caroux, 2023), also confirms the “importance of narrative closure and logical coherence for positive evaluations” (Krakowiak, 2024), and supports the finding that “visual stimulation can drive attention in the short term but is difficult to continuously convert into positive reviews” (Valentowitsch, 2024).

The innovations of the research lie in two points: (1) Proposing a “dual-core model” that can simultaneously explain emotional interaction and narrative effects, filling the gap of “separate research on FMV and dating simulations” (Hanapiah and Nasir, 2024); (2) Revealing the laws of community norms and discourse migration through netnography, indicating that audience evaluations will shift from “character love” to “romantic analysis and logical review” over time, and emotion and rationality are continuously reconstructed in interaction.

## 7 Conclusion

The findings indicate that emotional interaction and narrative structure are the two most salient dimensions in audience evaluation. As shown in Table 2, the four works display clear differences in overall satisfaction, with Knowledge, or Know Lady receiving the most positive evaluation and Fell in Love with Coser receiving the least positive evaluation. At the individual-comment level, Figure 2 further shows that the Pearson correlation between emotional classification and emotional scores is generally weak. Although some works reach statistical significance, the correlation coefficients remain low, and the combined sample also shows only a weak relationship. This suggests that emotional classification alone cannot fully explain variations in audience scores. These distributional findings and topic-modeling results are consistent with this view. As shown in Figure 3, works with higher audience satisfaction are more likely to contain more visible comments about emotional interaction and narrative experience. The LDA results in Table 4 and Figure 4 also show a similar trend. The main topics are clustered around emotional preference, endings, plot progression, and character relationships. These patterns suggest that audiences do not evaluate male-oriented live-action interactive films mainly on the basis of visual spectacle or technological novelty. More important seems to be whether the work can create believable emotional relationships and sustain a narrative structure that feels meaningful to the audience. Based on the netnographic analysis, this study also suggests a dual-core theoretical model of emotion and narrative, as shown in Figure 5. In this model audience evaluation is viewed as a result of interaction between two closely related dimensions. The emotional dimension includes the audiences’ attachment to characters, romantic imagination and emotional compensation; the narrative dimension includes the plot logic, narrative completeness and coherence of story development. In this sense, the success of male-oriented live-action interactive films is largely determined by the extent to which a work can mix emotionally engaging relationships with a coherent and persuasive narrative process. In summary, the long-term word-of-mouth and communication of interactive films rely on the resonance of “emotional authenticity” and “narrative closure.” Although visual presentation and technical implementation are necessary conditions, they are not sufficient to support positive evaluations alone; focusing creative and distribution resources on “perceivable emotional rewards” and “verifiable narrative causality” is the key path for the sustainable development of film-game integrated works.

## Data Availability

The original contributions presented in the study are included in the article/supplementary material, further inquiries can be directed to the corresponding author.
